# COVID-19 Intervention Scenarios for a Long-term Disease Management

**DOI:** 10.34172/ijhpm.2020.130

**Published:** 2020-07-26

**Authors:** Gudrun Wallentin, Dana Kaziyeva, Eva Reibersdorfer-Adelsberger

**Affiliations:** Department of Geoinformatics – Z_GIS, University of Salzburg, Salzburg, Austria.

**Keywords:** Scenario Analysis, Corona Virus, Pandemic, Agent-Based Model, Simulation, Containment

## Abstract

**Background:** The first outbreak of coronavirus disease 2019 (COVID-19) was successfully restrained in many countries around the world by means of a severe lockdown. Now, we are entering the second phase of the pandemics in which the spread of the virus needs to be contained within the limits that national health systems can cope with. This second phase of the epidemics is expected to last until a vaccination is available or herd immunity is reached. Long-term management strategies thus need to be developed.

**Methods:** In this paper we present a new agent-based simulation model "COVID-19 ABM" with which we simulate 4 alternative scenarios for the second "new normality" phase that can help decision-makers to take adequate control and intervention measures.

**Results:** The scenarios resulted in distinctly different outcomes. A continued lockdown could regionally eradicate the virus within a few months, whereas a relaxation back to 80% of former activity-levels was followed by a second outbreak. Contact-tracing as well as adaptive response strategies could keep COVID-19 within limits.

**Conclusion:** The main insights are that low-level voluntary use of tracing apps shows no relevant effects on containing the virus, whereas medium or high-level tracing allows maintaining a considerably higher level of social activity. Adaptive control strategies help in finding the level of least restrictions. A regional approach to adaptive management can further help in fine-tuning the response to regional dynamics and thus minimise negative economic effects.

## Background

The coronavirus disease 2019 (COVID-19) pandemic poses great challenges to governments around the world. Infection rates grew exponentially, which in some cases led to a breakdown of national health systems. To contain the virus, many countries took extensive lockdown measures. While the spread of the virus could be supressed this way within a couple of weeks, the lockdown caused great damages to the national economies and resulted in high rates of unemployment.

First hopes that the populations could develop herd immunity and thus quickly allow returning to normal economic and societal activities proved not feasible. Under controlled conditions of a flattened outbreak curve, it would take several years to get to infection rates of about 65% that are necessary for the natural containment of the virus by herd immunity.

Further, a vaccination cannot be expected earlier than about 2 years after the pandemic outbreak. Therefore, any relaxation of the lockdown poses the risk of a second outbreak. Strategies have to be developed to keep the outbreak within limits that does not incapacitate health systems, but at the same time allow the largest possible level of social and economic activity. The interventions have to be targeted so that hospitals can cope with the number of severe cases, especially in terms of intensive care unit beds.

Intervention measures either target at reducing contacts or reducing the transmission risk (hand washing, wearing face masks). The rigour of interventions taken in different countries range from rather soft measures relying on a set of voluntary recommendations to a full lockdown and surveillance regime. Possible strategies at the first sight appear like a choice between plague and cholera. On the one hand, measures can target the entire population of a country, which causes severe societal and economic impacts. On the other hand, these measures can be applied only to persons, who had been in contact with infected persons. While this option causes less impact on a general level, it raises severe privacy concerns.

Austria was amongst the European countries that took severe lockdown interventions relatively early in the outbreak and thus could overcome the first peak already by the end of March 2020. The Austrian government then announced a stepwise and closely controlled strategy to relax the lockdown measures and turn back to a “new normality.” In this phase, extensive testing aims to monitor the situation, based on which the government can quickly adapt their interventions.

As an important part of the counter measures, contact tracing of potentially infected persons is discussed complementary to the lockdown regulations. Some Asian countries like Singapore, Taiwan or South Korea proved highly successful in effectively containing the COVID-19 pandemic with digital surveillance methods and contact tracing.^1^

In Europe, contact tracing apps are discussed controversially.^2,3^ Apps need to meet related privacy policies and obligatory use is hardly accepted. However, such apps may play a decisive role in breaking chains of infection.^4^ In Austria, like in many other countries, a tracing application for smartphones was developed on behalf of the Austrian Red Cross. This “Stop Corona” app was rolled out by end of March 2020. It complements more traditional ways of contact tracing, most importantly interviews with newly infected that are conducted by health authorities.

Due to the lack of empiric knowledge regarding the new Corona virus, it proved extremely difficult for governments to make adequate decisions. A different mix of interventions at different pace were followed by European countries. The time lag between interventions taken and a measureable response in the infection rates was about 2 weeks, which is extremely large in the phase of the highly dynamic, exponential spread at the beginning of the outbreak. Without the possibility of quick feedback, the notion of “flying blind” through the crises was often cited in newspapers.

In this context, simulation modelling can be an important means to explore the effectiveness and the impact of interventions and thus help to minimise fatalities caused by the COVID-19 outbreak and trade off with economic damage, and privacy concerns. Agent-based modelling is a modelling approach that is specifically adequate to assess the dynamics of an epidemic in a specific region.^5^ Unlike mathematical models that need to be parametrised with empirical factors and rates at the population level see eg,^6^ agent-based models simulate how people interact and behave in geographic space. Agent-based modelling thus is especially well suited to explore alternative response strategies in a specific region.^7^

A number of ABMs were developed already in early phases of the outbreak, eg, to assess the role of travel activities in the Wuhan outbreak,^8^ to explore the impact of social structures in Italian’s hotspot region Lombardy,^9^ or to support decision-making for the government of Australia.^10^ Many of these early models re-parameterised models of other epidemic outbreaks like influenza,^11^ severe acute respiratory syndrome (SARS)^12^ or the measles.^13^

In this paper, we present a new, spatially explicit agent-based model the “COVID-19 ABM” to explore 4 different scenarios that represent the major alternatives for a long-term response strategy: (1) to continue the lockdown until the virus is regionally eradicated, (2) to slowly relax the lockdown to the maximum level without causing a second outbreak, (3) to slowly relax the lockdown and complement it with technology-supported contact tracing, or (4) to stepwise relax the lockdown controlled by an adaptive response strategy. If active cases exceed a given threshold some of the relaxations are taken back. The model is implemented for the city of Salzburg, but findings are largely independent from the study area.

## Methods

The COVID-19 ABM presented in this paper was developed based on 2 previously existing agent-based models. First, a sophisticated model of the mobility of citizens in the greater region of Salzburg city,^14^ and second a virus spread model from the GAMA modelling library.^15^ The virus model was extended, integrated with the mobility model and then calibrated with official case data for Salzburg ^16^. The model was implemented with the GAMA modelling environment.^17^ The source code of the “COVID-19 ABM (version 1.0.1)”^18^ is published on the CoMSES OpenABM modelling platform (https://www.comses.net/) under the open CC-BY-NC-SA licence.

The 2 main input parameters are the basic reproduction factor R0 and the daily contact rate. The sensitivity of these parameters was analysed within the ranges of their respective uncertainty. Finally, scenarios were designed and each scenario was simulated 6 times to assess the underlying uncertainty. Six repetitions were considered adequate, as the coefficient of variation^19^ converged already after the second repetition in all 4 scenarios and one repetition was quite time-demanding with 2 to 4 hours of computation time.

In the following, the model structure is described according to the overview section of the ODD protocol^20^ for reporting agent-based models.

### Purpose

The COVID-19 ABM aims to predict the qualitative behaviour of the COVID-19 epidemic dynamics for the greater region of Salzburg city. Specifically, by means of scenario testing, it aims to help assessing how containment interventions can allow a stepwise relaxation of the lockdown without risking a new outbreak.

### Entities, State Variables and Scales

The study area is the city of Salzburg with the adjacent municipalities, from where many people commute into the city to work, but also for shopping, recreation or other purposes. The region has about 200 000 inhabitants.

Infected persons, as well as potentially infected persons (“contacts”) are modelled explicitly as individual agents. The healthy, susceptible population, as well as the recovered, immune population is represented by a raster with a 250 m by 250 m grid resolution. Agents interact with the local population at the respective grid-cell. Finer grids for demographic data are neither available due to data privacy restrictions, nor necessary to model human activity patterns in very high resolution.^21^

A contact in the context of COVID-2 transmission according to the European Union^22^ and implemented in the Austrian “Stop Corona” app is defined as the physical proximity between 2 people of 2 m or less for at least 15 minutes. Accordingly, the time step resolution of this model was set to 15 minutes.

### Process Overview and Scheduling

**Figure 1:**
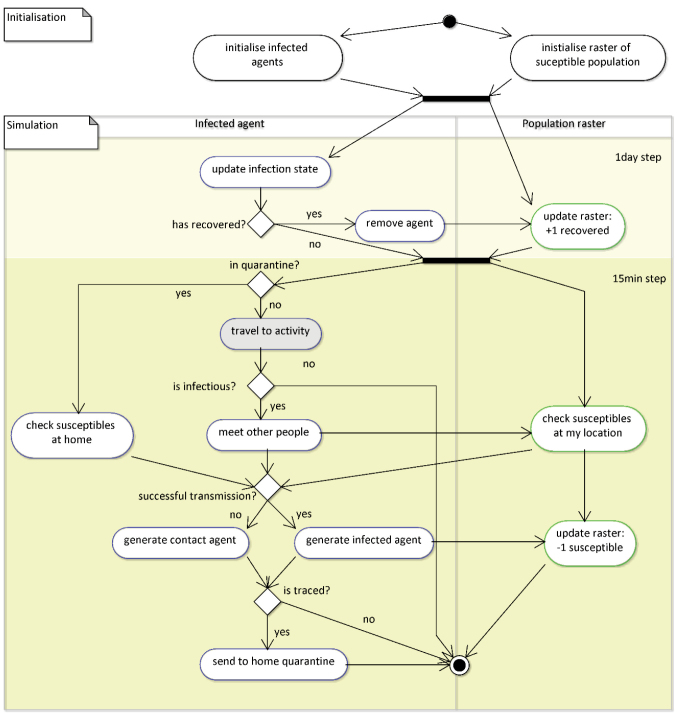
A UML Activity Diagram of the COVID-19 ABM. Abbreviations: UML, Unified Modeling Language; COVID-19, coronavirus disease 2019.

The Unified Modeling Language (UML) activity diagram of the model in Figure 1 provides an overview of the modelled processes. At each time step, an infectious agent locally passes on the virus to contact persons. The transmission probability is informed by the basic reproduction factor R0, which is reduced by local immunities at the cell or the facility at which the agent is located. In contrast to non-spatial models, infection probabilities thus depend on individual contacts and vary locally.

In the UML activity diagram, the mobility part of the model is lumped together to the “travel to activity” (shaded in grey) activity, whereas the virus part of the model is presented in full detail.

### Virus Model Part

The virus part of the model represents the infection process according to Ferretti et al^4^ as shown in Table 1.

**Table 1:** The Schedule of the Infection Process

**Day 1–Day 3**	Infected, but not infectious
**Day 4**	Infectious, without symptoms
**Day 5–day 15**	Symptomatic and infectious
**>Day 15**	Recovered

An important delay between new cases in the model and empirical data is the time between a person has developed first symptoms, until it is recorded as case in official databases. This results from the cumulated delays of reporting symptoms, response from health authorities, sample taking, analysis of samples and database entry. Especially in the peak phase of the outbreak, this delay was estimated to about ten days, which is comparable with experiences in other countries.^1,23^ This time lag had to be taken into account especially for calibration and validation by considering the infection date to be 10 days earlier from the report date of a positively tested person.

About half of the infections happen in the pre-symptomatic phase before a patient feels sick,^4^ and thus follow their usual daily activities. Most pre-symptomatic infections occur the day before symptom onset.^24^ In the model, this pre-symptomatic, infectious phase lasts for one day. The other 50% chance to pass on the virus stretches over the entire symptomatic phase.

Three further key assumptions were made. First, we assumed that there are no infections by asymptomatic (rather than pre-symptomatic) persons. Ferretti et al^4^ argue that while asymptomatic infections are common, forensic reconstructions have shown that asymptomatic persons rarely passed on the SARS-CoV-2 virus. Second, we assumed that recovered persons stay immune for some time. While this seems to hold true for a few months, it is not yet clear, whether protective immunity will last longer.^25^ Finally, the assumption was made that all quarantined persons as well as all symptomatic persons strictly stayed in home quarantine.

### Mobility Model Part

The mobility model part accounts for all activities of citizens that generate mobility within the greater region around the City of Salzburg. It is based on the “Bicycle model for Salzburg City” v2 that was implemented by one of the co-authors (DK). The model is calibrated with demographic data and mobility surveys. Of this model, only the explicit route calculation for bicyclists were taken out, so that all individuals are “teleported” between their daily activities. For further details of the underlying “Bicycle model,” see the fully documented model, which is published in the CoMSES computational model library.

### Input Data and Calibration

The 2 key input parameters for the virus model are the basic reproduction factor R0 and the average daily contacts before lockdown that are closer than 2 m and more than 15 minutes.

For the *basic reproduction factor R0* a default value of 2.75 is assumed. This factor describes the speed at which COVID-19 is able to spread in a particular population. As it characterises the “aggressiveness” of the virus as well as the social interaction level of a society, it cannot be simply adopted from empirical studies in other countries. Also, reported values in the literature differ widely. However, many studies agree on levels of R0 between 2.5 and 3.0.^26,27^

The average *contacts per day* before lockdown, are parametrised with a default value of 6.4 daily contacts. Mossong et al^28^ reported 7.95 daily contacts for Germany, 80% of these (6.4) lasted longer than 15 minutes and thus are relevant contacts for the spread of COVID-19. As Austria is comparable with the German culture, the COVID-19 ABM assumes 6.4 contacts per day before the lockdown.

However, if the COVID-19 ABM is to be transferred to other countries, it has to be noted that this value varies extremely across countries and cultures. In Europe contacts per day range between 8 in Germany up to 20 in Italy.^28^ For the extremely dense population of Singapore, Ooi et al^29^ report 38 contacts per case on average in Singapore for the SARS1 outbreak.

Further input data include data on population, mobility and the epidemics; see Table 2 for a detailed list.

**Table 2:** Overview of the Input Data That Were Used in the COVID-19 ABM

**Input Data**	**Value/Data Set**	**Source**
Initially infected persons on March 16, 2020	63 (extrapolated considering an initial 7-day reporting delay) with an exponential distribution of the attribute “days since infection”	Public data records, Authority for Public Health, Land Salzburg
Population (residents)	250 m × 250 m raster data	Salzburg bicycle model v. 2.0, from Statistik Austria
Facilities for activity (work places, shops, schools, etc)	Point data	Salzburg bicycle model v. 2.0, from diverse data sources
Mobility behaviour	Demographic probability distributions	Salzburg bicycle model v. 2.0, from mobility surveys

Abbreviation: COVID-19, coronavirus disease 2019.

Calibration was based on public data records. These were only available at an aggregate level of districts, not municipalities. So the number of active cases in the study region had to be extrapolated from case data and matched with population distributions.

### Sensitivity Analysis

A sensitivity analysis was conducted for the 2 critical parameters R0 and number of contacts before the lockdown. The aim of the sensitivity analysis especially targeted at qualitative changes in the infection dynamics. The scenario for sensitivity analysis was the low-level contact-tracing scenario, which appeared to be the most probable strategy at the time of writing this paper (April 2020). The default values were 2.5, 3.0, and 3.5 (default 2.5) for R0, and 5, 6.4, and 8 (default 6.4) for the daily contacts respectively.

### Validation

At the time this manuscript was written, the quality of available case data was bad. As stated before, county-level case numbers had to be disaggregated with help of population data. Further issues were low testing rates, large time lags between the infection and the day a case was reported and an initially dysfunctional Coronavirus hotline. Altogether, the situation demanded decision-making under severe uncertainty. Thus, rather than (over)fitting the model to – and validating the model with – uncertain data, we attempted to represent a structurally sound process model together with an uncertainty assessment for the key parameters.^30^ Insights from scenario results thus can be derived with respect to their qualitative behaviour, but any quantitative statement comes with large levels of uncertainty.

### Scenarios

For this paper, 4 scenarios were defined that represent alternative pathways and aim to assess their mid-term effect in terms of the dynamics of the epidemic.

*1. Continued lockdown “lockdown scenario”:* This scenario continues the initial, severe lockdown interventions with quarantine regulations for the entire population until regional eradication of the pandemics in the greater region of Salzburg City.

*2. Stepwise relaxation of the lockdown “relaxation scenario”:* In this scenario, the initially severe lockdown (March 16) is relaxed stepwise. It follows the intervention plan as communicated by the Austrian government in April 2020 (see Table 3): small shops open again (April 14), all shops open (May 1), small events are allowed (July 1).

**Table 3:** Parameterisation of Mid-term Intervention Scenarios to Contain the Spread of COVID-19

	**Continued Lockdown**	**Relaxation of Lockdown**	**Relaxation With Contact Tracing**	
	**Contacts Per Day**	**Contacts Per Day**	**Contacts Per Day**	**% Contacts Traced**
Before interventions	6.4	6.4	6.4	3%
16 March: start of lockdown	1.2	1.2	1.2	6% | 12% | 18%
14 April: small shops open	1.2	2.3	2.3	9% | 18% | 27%
1 May: all shops open	1.2	3.2	3.2	12% | 24% | 36%
1 July: small events allowed	1.2	5.0	5.0	13% | 26% | 39%

Abbreviation: COVID-19, coronavirus disease 2019.

*3. Relaxation of the lockdown paralleled with contact tracing “contact-tracing scenario”:* This third scenario builds on the previous relaxation of an initially severe lockdown, but parallels it with traditional and voluntary contact-tracing measures. Three sub-scenarios are computed for low, medium and high levels of contact tracing.

*4. Stepwise relaxation with monitoring and adaptive response “adaptive response scenario”:* This scenario was presented as the backup strategy by the Austrian government. If follows the “relaxation scenario.” However, the situation is closely monitored and whenever the number of active cases exceeds capacity-levels of intensive care units, some of the relaxation measures are taken back until numbers again fall under the threshold. The threshold level for the study area was estimated to be 200 active cases.

The relative reduction of the daily contacts during the lockdown was estimated from Google’s Community Mobility Reports (https://www.google.com/covid19/mobility/) for the county of Salzburg between February 23, 2020 and April 5, 2020. The impact of further relaxation of the lockdown is informed by the relative change of mobility for this period in reference countries. Especially Australia for the stage, when all shops are allowed to open again and mobility response to the quite liberal interventions in Sweden are taken as a reference for the last stage of lockdown relaxation on July 1, 2020. See Table 3 for a summary of parameters.

Data to estimate the share of successfully traced contacts before they get infectious are sparse. Ng et al^1^ provides evidence from the first 100 infected persons of the COVID-19 outbreak in Singapore, where 13% of contacts could be traced and isolated before they developed symptoms. For the parameterisation of the low-level contact-tracing scenario, it was assumed that contact tracing is conducted mainly with traditional methods of interviews, as it is also laid out in the official guidelines of the Austrian Ministry of Health. Given the enormous initial time lag between the time, when a patient develops the first symptoms and when this person is registered as a case, the number of effectively traced cases was probably low. However, as the COVID-19 epidemics proceeds, testing efficiency increases and awareness about the disease rises, the number of contact persons, who are quarantined before they get infectious will increase as well. On the other side, in the last step of relaxation, when public spaces are opened and small events are allowed, contacts with unknown people will increase and negatively impact the success of contact tracing.

Especially in terms of unknown contacts, Smartphone applications will likely further support traditional contact tracing efforts. However, even if 20% of the population downloaded the app and self-quarantine when prompted, these are independent events according to probability calculation and thus only would cover 4% of contacts. Taking all these considerations into account, we assumed that the percent of effectively traced contacts will rise from 3% to 13% of all potentially infectious contacts until July. In scenarios, where health authorities get support from the police and the use of the tracing app is combined with incentives this share may double to 26% or even triple to 39% (see Table 3 for further details).

## Results

**Figure 2:**
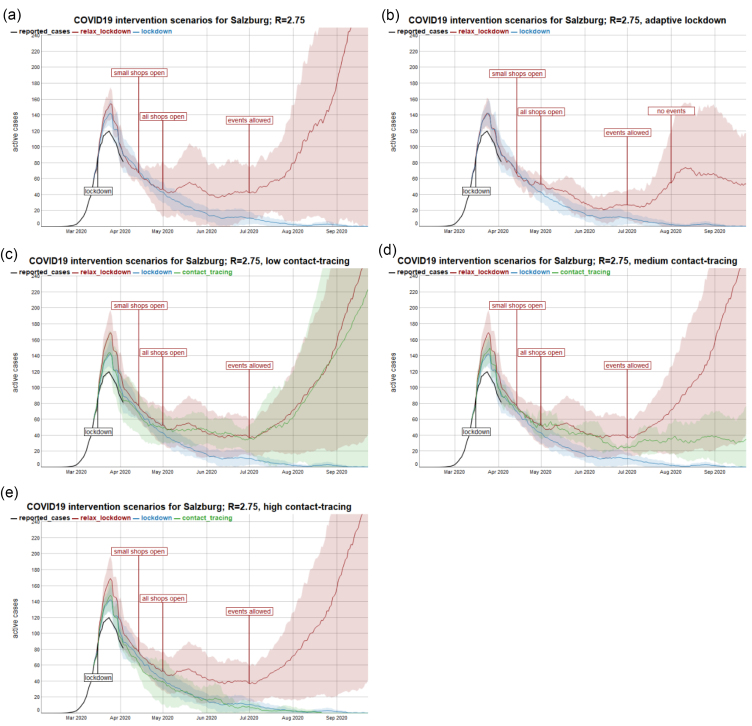
Mid-term Intervention Scenarios: (a) lockdown vs. stepwise relaxation of lockdown as communicated by the Austrian government, (b) monitoring and adaptive response to partially reverse relaxation, (c) – (e) stepwise relaxation of lockdown paralleled with contact tracing: (c) low (up to 13% of contacts successfully traced), (d) medium (26% traced), (e) and high level of contract tracing (39% of contacts successfully traced). Abbreviation: COVID-19, coronavirus disease 2019.

The alternative intervention scenarios after the lockdown result in very different outcomes. According to the simulation, the continuation of a strict lockdown can eradicate COVID-19, while a stepwise relaxation back to about 80% of previous contact rates results in a second outbreak (Figure 2a). However, due to the slow and stepwise relaxation, initial growth rates are slow and there is enough time to take back some of the relaxations to control the dynamics. A level of at least twice the social contacts as in the lockdown phase, which corresponds to an average of 3.2 daily contacts (“all shops open”) is sustainable (Figure 2b).

Contact tracing is a complementary measure that holds a potential for further increasing the average social activity to 80% of its previous level. However, the success of contact tracing depends on how widely it is deployed (Figure 2c-e). Low levels of contact tracing have only negligible effects (Figure 2c). Only with tracing rates of 26% of successfully traced and isolated contacts can stabilise contact rates at the 80% level compared to pre-epidemic times (Figure 2d). Tracing rates that track down 39% of contacts have the potential to help eradicate the virus in the study region (Figure 2e).

Although qualitative behaviour in terms of the general trend of a scenario is mostly stable, the uncertainty is high. Small stochastic variations during a simulation can result in a wide range of simulation outcomes for the same set of parameters. The shaded areas of the predictions in Figure 2 mark the 95% confidence interval of 6 scenario repetitions each. For the relax-lockdown scenario for example, the uncertainty even is qualitative, ie, it ranges from full control of the epidemic to a second outbreak.

**Figure 3:**
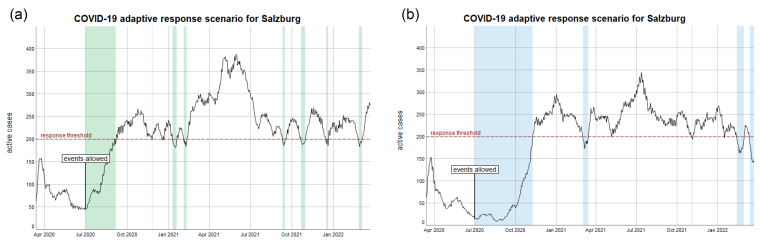
Long-term Simulation of the Adaptive Response Strategy. The periods of time that are colour-shaded visualise times with relaxed restrictions. Simulations (a) and (b) are 2 independent repetitions for the same scenario. They show that general oscillation patterns and value ranges are robust, but the timing of peak periods differs. Abbreviation: COVID-19, coronavirus disease 2019.

Figure 3 presents how an adaptive response scenario could unfold over the next 2 years until the availability of a vaccine can be expected. Each time a threshold of 200 active cases is reached, relaxations need to be taken back to prevent a second outbreak. In this specific scenario, the stage of “events allowed” with small events and opened museums is reverted to the lower level of “all shops opened.” According to this scenario, events would be allowed only during the rare and short periods, while most of the time they are restricted. Due to the powerful exponential growth, case numbers can oscillate with peaks that lie considerably above that threshold (386 for scenario a, 345 for scenario b).

Figure 3 shows this adaptive response scenario for 2 independent simulation runs. Stochastic variations result in a different pattern and pacing of the dynamics. At the end of the simulation in March 2022, 2 years after begin of the outbreak, 4.4% or 5.2% of the population are immune. After 3 years, about 7% of the population are immune, given that individual immunity does not decrease over time.

**Figure 4:**
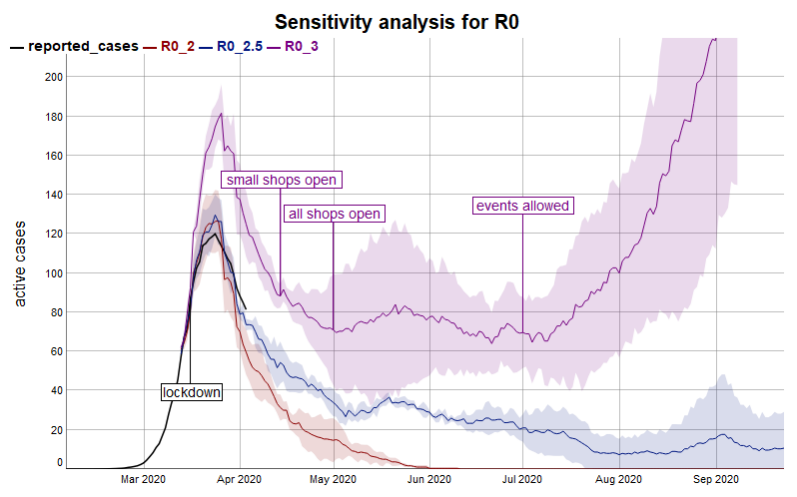
Sensitivity of the Epidemic Dynamics With Regards to R0; Based on 6.4 Daily Contacts and the Medium-Level Contact Tracing Scenario.

A caveat in the interpretation of the presented scenarios is the high sensitivity of the model with regards to R0 (Figure 4). The range for R0 between 2 and 3 that is given in the literature results in either quick and full eradication (R0 = 2.0), successful containment (R0 = 2.5) or exponential growth in a second outbreak (R0 = 3.0).

**Figure 5:**
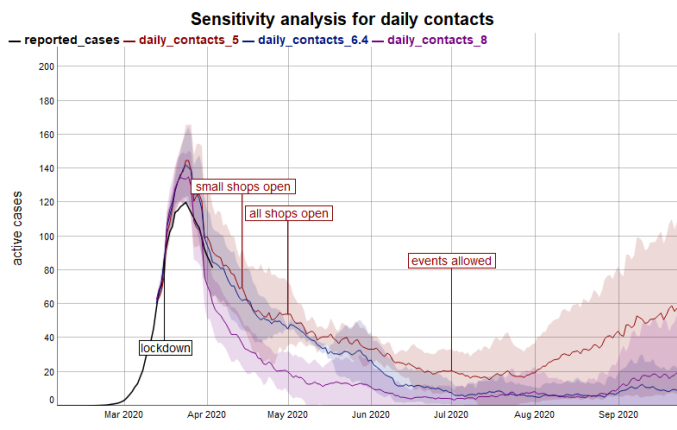
Sensitivity of Simulated Dynamics With Regards to the Number of daily contacts Before the Lockdown; Based on R = 2.5 and the Medium-Level Contact Tracing Scenario.

In contrast to R0, the model is not very sensitive for the parameter of daily contacts within the tested range of 5 to 8 contacts (Figure 5).

## Discussion

### The Added Value of Contact Tracing

Ng et al^1^ gives an insightful report of the first 100 COVID-19 patients in Singapore. Fifty-three percent of cases were detected through contact tracing. The majority of these 53% already have developed symptoms at the time, when officials isolated them and thus quarantine came (too) late. However, 13% were still identified in time. In reference to these data, the “low-level” contact tracing scenario was parameterised with up to 13% timely traced contacts.

Although ambitious, this rate could also be achieved in European countries as testing capacities increase, the interval from onset of symptoms to isolation of contacts decreases, and privacy issues of tracing apps get solved. Incentives to use the app and awareness of its value in gaining considerable relaxation of lockdown measures may help to reach a medium-level contact tracing. A smart combination of interview-based, traditional contact tracing to prevent misuse and a supporting use of the app to speed up the process may guide the way ahead.

### Good Thresholds for Adaptive Response

While adaptive response appears to be a smart strategy, a closer look at the adaptive response scenarios in Figure 2b and Figure 3 shows that the adaptive scenarios exhibit comparatively high case numbers that clearly exceed the intended threshold level. Further and counter-intuitively, this stressful healthcare situation does not come with much benefit for the economy. There are only short periods of relaxation in which restrictions could be taken back. However, for policy-makers it is hardly feasible to frequently change between imposing and relaxing restrictions, so that they will likely keep restrictive policies in place.

It can be intentional to keep case levels high, if the goal was to reach herd immunity. However, even under the optimistic assumption of a long-term immunity after recovery the adaptive response strategy only reached a population immunity rate of about 5% after 2 years.

Thus, the scenario suggests that the better choice would be to keep threshold levels low and immediately react to rising case numbers by taking back relaxation measures until a vaccination is available. With the same amount of restrictions for the economy, a lower threshold has obvious benefits for the healthcare system.

### Regional Containment: A Way Forward

Another insight can be derived from considering the 2 independent simulations of the adaptive response strategy in Figure 3. They show the same oscillation pattern, but numbers peak at different times. This can be interpreted as the progress of the epidemics in 2 regions that are not strongly linked. Global measures based on case numbers that are aggregated across regions may be adequate for the one, but not the other region. Numbers may start rising exponentially in a particular region, although aggregate averaged values still decrease. A global relaxation of the lockdown would then lead to local growth dynamics in that particular regions that are hard to control. Therefore, adaptive control and response strategies should be designed regionally. This could include individual agreements with local partners to apply response strategies quickly and efficiently reduce the social interaction level in that particular region, if necessary. Such partners could be for example large or mid-sized companies that switch to home office, but also youth associations or the public sector.

### The Role of Models. What They Can Do and What They Cannot

Wynants et al^31^ reviewed early published COVID-19 models, based on which they identified a need for more rigorous modelling. In acute situations like the pandemic outbreak rapid model development is critical. However, this also implies the risk of highly uncertain or even flawed models. Regan et al^30^ thus postulates for such situations to fully acknowledge and treat uncertainty and thus avoid ill-informed decision-making. Meeting this postulation is even more important, as we now transition into the “new normality,” the second and less acute phase of the epidemics.

The findings of this research are based on modular model development of previously tested parts, sensitivity testing of critical parameters and uncertainty assessment. While agent-based simulation models are well suited to understand, how systems behave in response to a change in the behaviour of individuals, the accurate prediction of numbers is not a strongpoint of this approach. Thus, like other agent-based models, the COVID-19 ABM is a “tool to think with” rather than a precise forecast. Further, there lies a value in designing the model per se. Reliable and robust statements can be made about the general behaviour, for example that oscillations clearly exceed the threshold set in the adaptive scenario, that large stochastic differences can be expected in independent regions, or that medium-level contact tracing can considerably help to reduce general lockdown restrictions.

## Conclusion

Three main insights for mid-term intervention strategies can be derived from the scenarios with high rates of confidence:

First, the effect of low-level contact tracing is negligible, but medium or high-level tracing of about 15% to 20% of contacts can be expected to help relaxing general restrictions considerably without risking a second outbreak.

Second, adaptive control and response management should not be applied on a global level, because the dynamics in individual regions can differ widely. This management should rather target individual regions that have strong within-region mobility, but have less physical mobility into other regions.

Third, threshold levels for adaptive management should be kept low. Higher levels lead to higher case (and mortality) numbers, but nevertheless do not allow for longer periods of relaxation. Aiming at high levels should only be considered, if successful development of a vaccine cannot be expected within the next 4 or 5 years and the aim is to reach herd immunity.

## Ethical issues

An ethical approval was not necessary, because we only used aggregated case numbers that were openly published by the Austrian Ministry of Health.

## Competing interests

Authors declare that they have no competing interests.

## Authors’ contributions

GW was the lead author. She designed the research, programmed the model, conducted the analyses and wrote the manuscript. DK contributed to programming the model, she especially adapted the mobility model part and critically reviewed the manuscript. ERA contributed to the conceptual model design and critically reviewed the manuscript.

## Key Messages

**Implications for policy makers**

- Supports policy-makers in assessing the success of alternative containment strategies after the coronavirus disease 2019 (COVID-19) lockdown phase.
- Results suggest that the stepwise, controlled relaxation of lockdown and its long-term management, should be managed at a regional level.
- Contact tracing gets only effective above 25% of contacts isolated within the first 3 days. However, then it allows for social and economic activity at high levels.

**Implications for public**

Scenario research shows that there are 2 pathways to successfully manage coronavirus disease 2019 (COVID-19): First, general restrictions for everybody that aim to keep social life to about half of its previous level. Second, rapid isolation of those, who have been in close contact with an infected person. The latter approach potentially allows that most people can return to an (almost) normal social life. However, contact tracing needs to be fast and isolate contacts, before they feel sick. This needs the contribution of everybody:

- Keep a diary with whom you had close contact, or use the available contact tracing apps.
- If you feel sick, call the doctor immediately, do not wait for “tomorrow.”
- If you are tested positive: inform all close contacts of the previous 2 days immediately.
- If you are a contact yourself and you are asked to isolate, avoid any social contact, even if you do not feel sick.
